# Acute generalized exanthematous pustulosis during apalutamide treatment in a patient with prostate cancer

**DOI:** 10.1002/iju5.12525

**Published:** 2022-08-16

**Authors:** Tomoko Honda, Yoichiro Tohi, Yo Kaku, Nachino Kimura, Takuma Kato, Reiji Haba, Teruki Dainichi, Mikio Sugimoto

**Affiliations:** ^1^ Department of Urology, Faculty of Medicine Kagawa University Kagawa Japan; ^2^ Department of Dermatology, Faculty of Medicine Kagawa University Kagawa Japan; ^3^ Department of Diagnostic Pathology, Faculty of Medicine Kagawa University Kagawa Japan

**Keywords:** acute generalized exanthematous pustulosis, adverse event, apalutamide, skin rash

## Abstract

**Introduction:**

Acute generalized exanthematous pustulosis is a type of severe cutaneous drug adverse reaction. While apalutamide is known for its high incidence of cutaneous adverse events, it remains unknown whether acute generalized exanthematous pustulosis can develop during apalutamide treatment.

**Case presentation:**

A 72‐year‐old man with metastatic castration‐sensitive prostate cancer developed small erythema on the face and trunk after 41 days of apalutamide treatment. Three days later, apalutamide was discontinued. However, 9 days after the discontinuation of apalutamide, the patient had a high fever with hemodynamic instability and showed diffuse erythema throughout the body with numerous small pustules. Skin biopsy revealed subcorneal and intraepidermal pustules admixed with many eosinophils, which led to the diagnosis of acute generalized exanthematous pustulosis. The skin rash improved in 14 days with systemic corticosteroid administration.

**Conclusion:**

We present the first case of a skin rash with clinical features of acute generalized exanthematous pustulosis during apalutamide treatment.

**Abbreviations & Acronyms**
AGEP: acute generalized exanthematous pustulosis
DRESS: drug rash with eosinophilia and systemic symptoms
HE: hematoxylin–eosin
ICU: intensive care unit
mCSPC: metastatic castration‐sensitive prostate cancer
mPSL: methylprednisolone
TEN: toxic epidermal necrolysis

**Keynote message**

AGEP is a type of severe cutaneous drug adverse reactions. Apalutamide is known for its high incidence of cutaneous adverse events. We present a case of skin rash presenting clinical features of AGEP with hemodynamic instability during apalutamide treatment.

## Introduction

Apalutamide is an androgen receptor targeting agents approved for the treatment of nonmetastatic castration‐resistant prostate cancer and metastatic castration‐sensitive prostate cancer (mCSPC).^1^, ^2^ Skin rash is one of the apalutamide‐associated adverse events and has been reported to be especially frequent in the Japanese population.^3^ There have been rare reports of the development of toxic epidermal necrolysis (TEN) in severe drug eruptions.^4^, ^5^

Acute generalized exanthematous pustulosis (AGEP) is one of the severe cutaneous drug adverse reactions with a fatal clinical course in up to 3.5% of cases.^6^ Typically, AGEP is characterized by high fever and rapid onset of edematous erythema and pustules with increased neutrophils without involvement of internal organs^7.^ It remains unknown whether AGEP can develop during the treatment with apalutamide. Here, we present a case of skin rash with clinical features of AGEP during apalutamide treatment.

## Case presentation

**Fig. 1:**
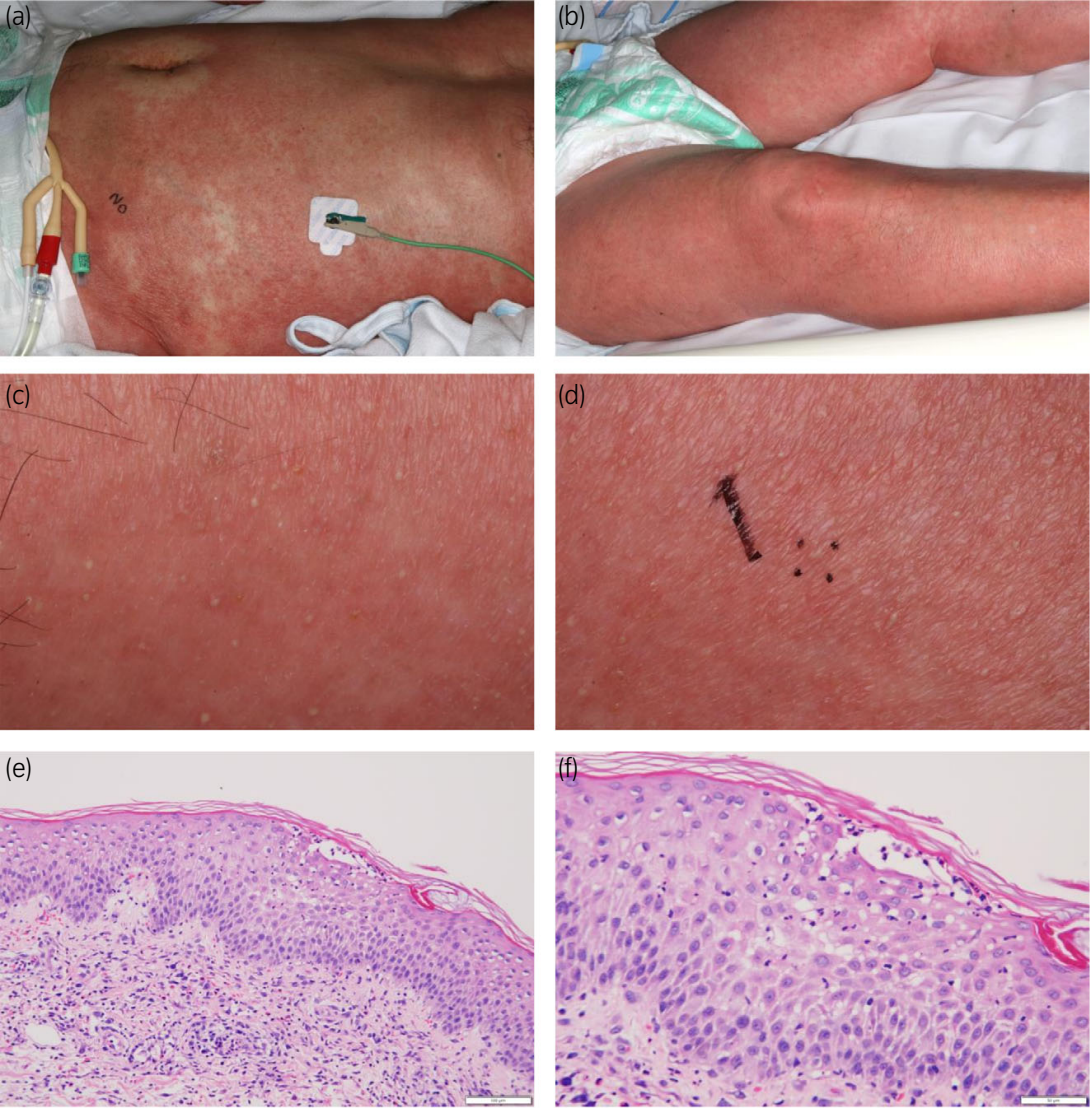
Clinical and histological features: Skin rash on both body (a) and legs (b). Edematous erythema with small pustules (c). A biopsy skin specimen of a pustule (marked) obtained from the forearm (d). Histological examination of the biopsy revealing perivascular and interstitial infiltration of lymphocytes and eosinophils in the dermis hematoxylin–eosin (HE) staining, ×20 (e). The subcorneal and intraepidermal pustules mixed with many eosinophils. HE, ×40 (f).

**Fig. 2:**
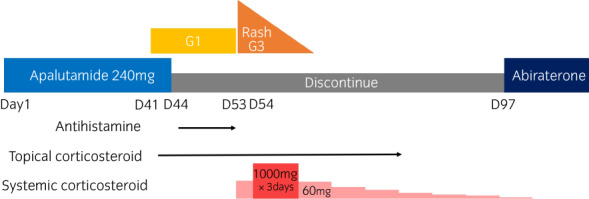
Clinical course of our case.

A 72‐year‐old male mCSPC [prostate‐specific antigen, 89.8; Gleason score, 5 + 5; cT3aN0M1(multiple bone metastasis)] patient with histories of transverse colon cancer, chronic glomerulonephritis, cerebral infarction (no aftereffects) and no allergies was initially administered apalutamide orally (240 mg/day) with a gonadotropin‐releasing hormone antagonist. The patient's height, weight, and body mass index were 162.0 cm, 57.7 kg, and 23.8 kg/m^2^, respectively. No new drugs other than apalutamide were administered. Forty‐one days after the initiation of apalutamide, the patient developed multiple, small erythema on the face and trunk with no high fever. Topical corticosteroid administration was not effective. Three days later, apalutamide was discontinued and oral antihistamine was administered on the order of the attending urologist. Nine days after the discontinuation of apalutamide, the patient had a fever of 38.1°C and his blood pressure decreased to 77/50 mmHg. The edematous erythema spread to the body, both forearms, and both legs (Fig. 1a,b). Generalized edema also appeared and multiple small pustules developed within the erythema (Fig. 1c,d). Mucosal erosion and Nikolsky's sign were not found. The erythema covered more than 30% of the body's area and was classified as grade 3 according to the National Cancer Institute Common Terminology Criteria for Adverse Events, version 5.0. Laboratory data showed slightly elevated levels of C‐reactive protein, 3.30 mg/dL; white blood cells, 11,610/μL; neutrophils, 8,816/μL; and eosinophils, 8.5% and normal liver function. No atypical lymphocytes were found. Echocardiography showed no cardiac dysfunction. The drug‐induced lymphocyte stimulation test was negative for apalutamide; however, no other drugs were changed or added except for apalutamide. AGEP was the most suspected diagnosis because of high fever and acute onset of edematous erythema and pustules with increased neutrophils without involvement of internal organs. Skin biopsy revealed subcorneal and intraepidermal pustules admixed with many eosinophils (Fig. 1e,f), which confirmed AGEP. However, drug reaction with eosinophilia and systemic symptoms (DRESS) was also listed to the differential diagnosis, with a score of 4 (probable DRESS) on the RegiSCAR scoring system.^8^ The patient required intensive care unit (ICU) management because his hemodynamic instability was not improved by transfusions. All medications were discontinued and 60 mg methylprednisolone (mPSL) was administered. The next day, 1,000 mg mPSL pulse therapy was initiated for 3 days and the patient's hemodynamics and fever stabilized. The patient was discharged from the ICU after 4 days. The skin rash did not progress and gradually improved to grade 1 on the 14th day after admission. All drugs except apalutamide were resumed and systemic mPSL was tapered. As a sequential treatment and because the patient was considered as high‐risk mCSPC according to the LATITUDE criteria, abiraterone was started on day 53 after the discontinuation of apalutamide (Fig. 2).^9^ The skin rash has not recurred.

## Discussion

We should consider that severe cutaneous drug adverse reactions can occur during treatment with apalutamide. Most of the apalutamide‐associated skin rashes can be manageable with dose reduction or discontinuation of apalutamide.^10^, ^11^ There are previous reports of TEN, during apalutamide treatment.^4^, ^5^ Our case was also different from Stevens–Johnson syndrome and TEN in the absence of mucosal findings and epidermal necrosis.^7^ In our case, the skin rash worsened persistently after the discontinuation of apalutamide, and the edematous erythema with small pustules spread throughout the body concurrent with high fever and hemodynamic instability. This clinical course required a differential diagnosis of severe drug eruptions such as AGEP and DRESS. Typically, AGEP differ from DRESS in terms of presence of significant generalized pustules and absence of other organ dysfunction.^7^, ^12^ Thus, we believe that our case was basically AGEP because of its clinical course, the phenotype of this rash, and the histology of skin biopsy. However, there have been several reports of DRESS with AGEP features.^13^, ^14^ In principle, the treatment of AGEP is discontinuation of the obviously causative drug, which leads to spontaneous resolution within a relatively short time frame.^7^ Systemic corticosteroid treatment is usually not necessary because AGEP has a good prognosis compared to other severe drug eruptions.^11^ However, the pathological results of skin biopsies generally require time to be definitive, and in our case, there were overlapping diagnoses of DRESS and AGEP. In cases where it is difficult to differentiate AGEP from other severe drug eruptions that may lead to fatal outcomes, we should not hesitate to use systemic corticosteroid therapy in clinical practice. In our case, corticosteroid pulse therapy was used because of the difficulty in differentiating AGEP from DRESS, which has a mortality of up to 20%.^12^

Another key finding was the worsening of the skin rash after the discontinuation of apalutamide. In our case, after the appearance of skin rash, apalutamide was not immediately discontinued, and 9 days after the discontinuation of apalutamide, the skin rash with clinical features of AGEP developed. The mechanism of the worsening of the skin rash, even after the discontinuation of apalutamide, was speculated to be due to the long half‐life of apalutamide (110–231 hours).^15^ Reportedly, the duration from the onset of skin rash to the discontinuation of apalutamide was longer in patients with the severe skin rash than in those with mild skin rash.^16^ Thus, we may consider the possibility that the skin rash may worsen after the discontinuation of apalutamide.

In conclusion, we present the first case of AGEP developing during apalutamide treatment.

## Author contributions

Tomoko Honda: Writing – original draft. Yoichiro Tohi: Conceptualization; writing – review and editing. Yo Kaku: Conceptualization; writing – review and editing. Nachino Kimura: Writing – review and editing. Takuma Kato: Writing – review and editing. Reiji Haba: Writing – review and editing. Teruki Dainichi: Conceptualization; writing – review and editing. Mikio Sugimoto: Supervision.

## Conflict of interest

MS has received lecture fees from Janssen Pharmaceutical K.K.

## Approval of the research protocol by an Institutional Reviewer Board

Not applicable.

## Informed consent

Informed consent was obtained from the patient for the publication of this case report and accompanying images.

## Registry and the Registration No. of the study/trial

Not applicable.
